# Evaluation of the Clinical Value of KL-6 and Tumor Markers in Primary Sjögren’s Syndrome Complicated with Interstitial Lung Disease

**DOI:** 10.3390/jcm12154926

**Published:** 2023-07-27

**Authors:** Fengqin Wei, Xinran Zhang, Shengnan Yang, Jing Geng, Bingbing Xie, Yanhong Ren, Huaping Dai

**Affiliations:** 1 Department of Pulmonary and Critical Care Medicine, China-Japan Friendship Hospital, Capital Medical University, Beijing 100029, China; 2 National Center for Respiratory Medicine, National Clinical Research Center for Respiratory Diseases, Institute of Respiratory Medicine, Chinese Academy of Medical Sciences, Beijing 100029, China; 3 Department of Pulmonary and Critical Care Medicine, Qingdao Municipal Hospital, Qingdao 266011, China; 4 Department of Clinical Research and Data Management, Center of Respiratory Medicine, China-Japan Friendship Hospital, Beijing 100029, China; 5 Department of Pulmonary and Critical Care Medicine, Tianjin Chest Hospital, Tianjin 300222, China

**Keywords:** primary Sjögren’s syndrome, interstitial lung disease, KL-6, CEA, CA153

## Abstract

Objective: primary Sjögren’s syndrome (pSS) is an autoimmune disease, of which the most common complication is interstitial lung disease (ILD). This study aimed to analyze the clinical value of Krebs von den Lungen-6 (KL-6), carcinoembryonic antigen (CEA), and carbohydrate antigen 153(CA153) in patients with pSS complicated with ILD (pSS-ILD), given that only few studies have evaluated this. Methods: This is a cross-sectional study. Serum KL-6 levels (U/mL) were measured using chemiluminescence immunoassay, and concentrations of serum tumor markers were determined using the immunofluorescence method in 64 cases of pSS-ILD (pSS-ILD group), 23 cases without ILD (non-ILD group), and 45 healthy controls. The correlation between KL-6 and tumor markers as well as lung function was analyzed, and the factors that were associated with pSS-ILD were screened. Results: The serum KL-6 was more abnormally increased in patients with pSS-ILD, and the serum KL-6, CEA, carbohydrate antigen 125 (CA125), and CA153 levels were significantly higher in the pSS-ILD group than in the non-ILD and healthy control groups (*p* < 0.05). KL-6, CEA, and CA153 were negatively correlated with forced vital capacity (FVC%), forced expiratory volume in 1 s (FEV1%), total lung capacity (TLC%), and diffusing capacity for carbon monoxide (DLCO%) (all *p* < 0.05). Multivariate logistic analysis showed that KL-6 was an independent factor associated with pSS-ILD. Conclusions: In conclusion, we evaluated the association between clinical values of KL-6, tumor markers, and pSS-ILD, and found that KL-6 and tumor markers such as CEA, CA153, and CA125 in patients with pSS-ILD were higher than in patients with non-ILD, and KL-6 was more abnormally increased and significantly associated with ILD development in patients with pSS.

## 1. Introduction

Sjögren’s syndrome (SS) is an autoimmune disease with multiple organ involvement. Our previous study showed that connective tissue disease-related interstitial lung disease (CTD-ILD) is the second most common interstitial lung disease (ILD) after idiopathic pulmonary fibrosis (IPF, 24.1%). Of which, primary Sjögren’s syndrome (pSS) related ILD is one of the most common in Chinese ILD patients [1]. Other studies reported that the prevalence of ILD in patients with pSS is approximately 20% and that it is a severe complication of pSS. It is the main cause of increased mortality in patients with pSS [2,3]. However, given that patients in the early stage of pSS complicated with ILD (pSS-ILD) may be asymptomatic, the diagnosis may be missed. Since disease progression leads to decreased quality of life and increased mortality, early diagnosis and disease assessment are crucial. Biomarkers are biological observations that can substitute for clinically relevant endpoints or intermediate outcomes that are difficult to observe and can predict disease occurrence. It is easier and less costly to use clinical biomarkers than to directly measure final clinical end points, and biomarkers are usually measured in a shorter period of time. They can be used for disease screening, diagnosis, monitoring, and prognosis evaluation [4].

Krebs von den Lungen-6 (KL-6) is a mucin-like glycoprotein that has profibrotic and anti-apoptotic effects on lung fibroblasts. When subjected to an inflammatory storm, disulfide bonds near the epithelial membrane surface of type II AECs might be disrupted, and KL-6 might eventually diffuse into the lung epithelial lining fluid and blood stream. KL-6 with predictive value could predict who was more likely to develop progressive fibrosis and reflect some of the adverse consequences of ILD [5]. Serum KL-6 levels can reflect the severity of the alveolar epithelial injury, and the role of KL-6 as a biomarker for ILD has been reported [6]. Our group conducted a multicenter study previously in three leading hospitals of ILD in China and determined the diagnostic and prognostic values of serum KL-6 levels in Chinese patients with ILD. The serum KL-6 levels in patients with ILD were significantly elevated compared with patients with non-ILD pulmonary diseases or healthy individuals. In addition, after treatment, the serum KL-6 levels significantly declined in patients with improved ILD, indicating that as a biomarker of ILD, KL-6 is not only related to the diagnose of ILD but also related to the prognosis of patients [7]. A study by Choi MG et al. showed that the relative change in KL-6 over 1 week was useful for predicting in-hospital mortality in patients with AE-ILD. Significant changes in KL-6 levels help to indicate poor prognosis in patients with AE-ILD [8]. In 2021, we detected the levels of KL-6 in patients with IPF and found a significant elevation of KL-6 in IPF patients compared with other ILD and healthy controls. KL-6 is a useful noninvasive biomarker in distinguishing IPF from other ILD and evaluating disease severity and prognosis [9].

Serum KL-6 level has the potential to be used as a diagnostic marker for a variety of CTD-ILD. Hanaoka et al. [10] evaluated the role of serum KL-6 in patients with ILD complicated with polymyositis/dermatomyositis (PM/DM). The study validated the usefulness of serum KL-6 as a diagnostic marker for ILD in PM/DM. The baseline serum KL-6 level in patients with ILD was significantly higher than in those patients without ILD. However, serum KL-6 was not a short-term disease activity biomarker for ILD in PM/DM, but it was a long-term disease activity biomarker. In a clinical trial of ILD associated with systemic sclerosis, serum KL-6 levels were found to be associated with ILD severity and decreased with immunosuppressive therapy [11]. Lee et al. [12] confirmed that the elevated serum KL-6 levels in patients with CTD-ILD is positively correlated with computed tomography (CT) grade and negatively correlated with forced vital capacity (FVC) and diffusing capacity for carbon monoxide (DLCO), suggesting that elevated serum KL-6 levels are reflective of the severity of CTD-ILD. Furthermore, Ma et al. [13] included four groups of patients for study, which were the CTD-ILD group, CTD group, pulmonary infection group, and healthy controls. The serum KL-6 level in the CTD-ILD group was significantly higher than in the other three groups. The serum KL-6 level in the CTD-ILD group was positively correlated with the severity of HRCT disease, but negatively correlated with DLCO. The study shows that serum KL-6 level can be used as a potential diagnostic indicator of CTD-ILD.

Tumor markers are commonly used for the diagnosis and screening of tumors. In the early stages of lung cancer, breast cancer, pancreatic cancer, ovarian cancer, and other cancers, serum carcinoembryonic antigen (CEA), carbohydrate antigen 153(CA153), CA19-9, and CA125 levels may increase, which may also decrease after treatment, demonstrating their utility as markers of therapeutic efficacy. With in-depth understanding, the utility of tumor markers is no longer limited to the tumor type, and the usefulness of tumor markers has been reported in patients with ILD. Our group investigated serum tumor marker levels in ILD and found that serum CEA and CA125 levels were significantly higher in ILD patients than in patients with other lung diseases such as chronic obstructive pulmonary diseases, pneumonia, bronchial asthma, and ILD patients with elevated serum CEA and CA125 levels showed higher risk of cancer, indicating that serum CEA and CA125 levels may be a marker of cancer in ILD patients [14]. Jin et al. [15] showed that elevated CEA and CA19-9 levels could reflect the severity of CTD-ILD and could be used as its biomarkers. Wang T et al. [16] found that elevated CA125 levels can be used to evaluate the possibility of ILD in patients with rheumatoid arthritis (RA). Tumor markers CA153, CA125, and CA199 were increased in RA-ILD patients compared with RA without ILD patients. ROC curve analysis showed that CA125 was moderately correlated with RA-ILD. Yang Y et al. [17] discovered that CA125, CA153, and cytokeratin-19 fragment (CYFRA21-1) were increased in DM patients with ILD compared with DM patients without ILD. The area under the curve (AUC) of CYFRA21-1 for predicting ILD was 0.745. The AUC of CA125 combined with CYFRA21-1 was 0.801. Thus, the author draws a conclusion that elevated serum levels of CA125, CA153, and CYFRA21-1 could indicate the accompanying ILD in patients with DM. Shi et al. [18] showed that CA153 had the best diagnostic value in those tumor markers for pSS-ILD without malignancy. One recent study confirmed the levels of KL-6 and tumor markers greatly aided RA-ILD identification [19].

However, relatively few studies have evaluated the clinical value of KL-6 and tumor markers in patients with pSS-ILD. Therefore, this study aimed to evaluate the clinical value of KL-6 and tumor markers in patients with pSS-ILD and explore their association.

## 2. Methods

### 2.1. Study Subjects

A cross-sectional study was conducted in the National Center for Respiratory Medicine, China–Japan Friendship Hospital, from 1 January 2017 to 31 December 2020. This study was approved by the Medical Ethics Committee of the China–Japan Friendship Hospital. Written informed consent was obtained from all patients to collect patient data and biological samples. This study included 64 consecutive patients with pSS-ILD (pSS-ILD group) who were admitted to the hospital and diagnosed by a multi-disciplinary team (MDT). The patients suspected of having ILD undergo a standard investigation protocol including clinical signs, chest HRCT, lung function, bronchoalveolar lavage (BAL), and/or transbronchial lung biopsy (TBLB) or transbronchial lung cryobiopsy (TBLC), and the autoimmune antibodies for CTD, tumor markers and KL-6 as usual. A total of 23 patients without ILD and 45 healthy volunteers were also included as controls.

pSS was diagnosed on the 2016 American College of Rheumatology/European League for Rheumatology pSS classification criteria. Patients were excluded if they met the following criteria: (1) have evidence of other CTD diagnoses, such as systemic sclerosis, polymyositis/dermatomyositis, rheumatoid arthritis, systemic lupus erythematosus, and mixed CTD; (2) ILD caused by other causes, including exposure to drugs, toxins, livestock, and occupational environment; and (3) have evidence of chronic obstructive pulmonary disease, heart failure, acute pulmonary infection, respiratory malignancy, and pulmonary embolism.

### 2.2. Data Collection

The demographic and clinical data were collected, as well as chest HRCT images, lung function (percentage of predicted forced expiratory volume in 1 s [FEV1%], percentage of predicted forced vital capacity [FVC%], percentage of predicted total lung capacity [TLC%], percentage of predicted carbon monoxide [DLCO%]) and laboratory findings.

Fasting blood was collected from all subjects. Serum KL-6 levels were determined by the chemiluminescence immunoassay (Shenzhen, China) with the normal reference value of <500 U/mL. Serum CEA, CA19-9, CA125, and CA153 levels were determined by the Roche Hitachi Cobas E601 module (Hitachi, Tokyo, Japan) using the immunofluorescence method. The normal ranges used were CEA < 5.00 ng/mL, CA19-9 < 27.00 U/mL, CA125 < 35.00 U/mL, and CA153 < 18.00 U/mL.

### 2.3. Statistical Analysis

All methods were carried out in accordance with relevant guidelines and regulations. Continuous variables with normal distribution were presented as mean ± standard deviation, and variables with skewed distribution were presented as median (interquartile range, IQR). Clinical characteristics and biomarkers levels in different groups were compared using Student’s *T*-test or the Mann–Whitney U test (for continuous data) or chi-square test (for categorical data). Spearman’s correlation coefficient was used to analyze the correlation between biomarkers and lung function. The receiver operating characteristic (ROC) curve was used to analyze the clinical value of biomarkers in the diagnosis of pSS-ILD. Logistic regression analysis was used to determine the risk factors of pSS-ILD. SPSS26 and GraphPad Prism8 were used for statistical analysis and data plot. *p* < 0.05 was considered as statistically significant.

## 3. Results

The baseline characteristics of the study participants are shown in Table 1. There were 64 pSS patients with ILD, with 44 cases of them having the diagnosis of ILD earlier than pSS, 14 having ILD and pSS simultaneously, and 6 having ILD later than that of pSS. No significant differences were found in the demographic data and other baseline characteristics among the three groups. The levels of serum KL-6 were normal in patients without ILD, but significantly increased in patients with pSS-ILD compared with non-ILD and healthy controls (*p* < 0.01). All the serum levels of CEA, CA19-9, CA125, and CA153 in pSS patients with ILD were higher than that in patients without ILD and healthy controls (*p* < 0.01) but were still in normal ranges. Immunoglobulin G (IgG) level was also higher in patients with pSS-ILD (*p* < 0.05). Regarding lung function test results, there were no significant differences in FEV1%, FVC%, and TLC% between the pSS-ILD and non-ILD groups (*p* > 0.05), whereas DLCO% was lower in the pSS-ILD group (*p* < 0.05).

### 3.1. Diagnostic Value of KL-6 and Tumor Markers for pSS-ILD

**Figure 1:**
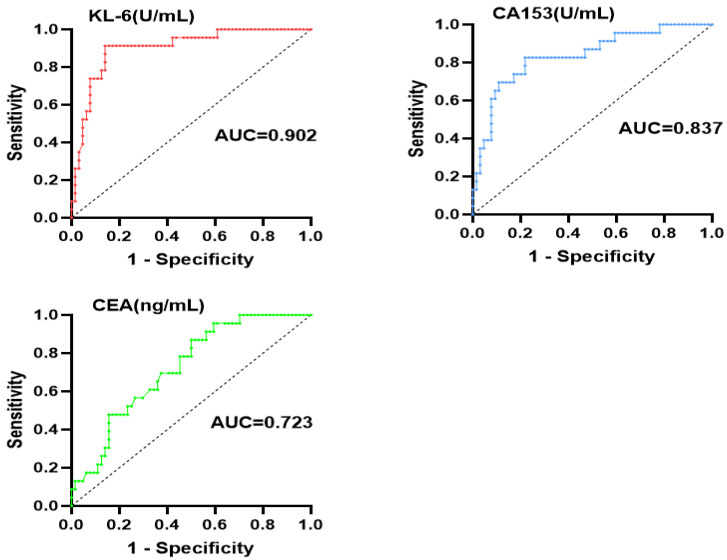
AUC of KL-6, CA153, and CEA for the diagnosis of pSS-ILD. AUC-area under the curve; pSS-ILD-primary Sjögren’s syndrome (pSS)-associated interstitial lung disease (ILD).

**Figure 2:**
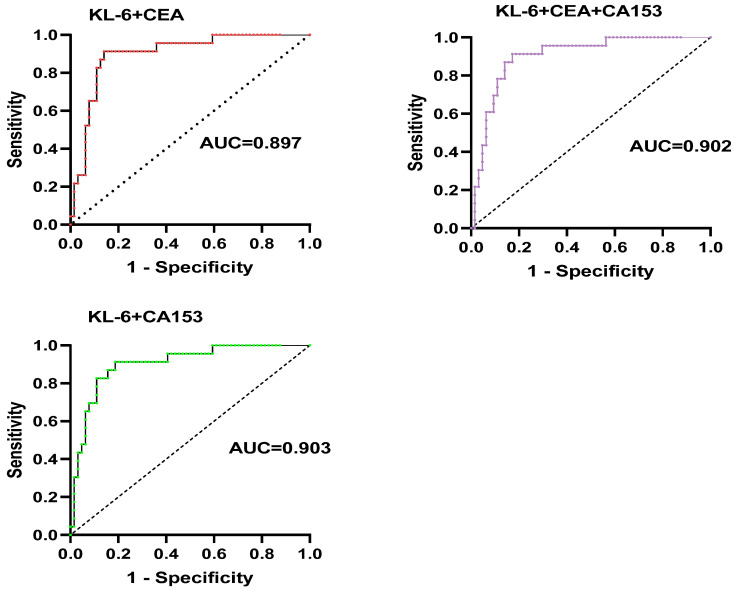
AUC of KL-6 combined with CEA and CA153 for the diagnosis of pSS-ILD.

ROC curve analysis showed that the AUC of KL-6, CEA, and CA153 were 0.902 (95%CI: 0.830–0.974), 0.723 (95%CI: 0.613–0.834), and 0.837 (95%CI: 0.738–0.936), respectively, and the corresponding cut-off values were 427 U/mL, 3.14 ng/mL, and 14.62 U/mL, respectively (Figure 1). ROC curves of multiple indicators were compared. The AUC of KL-6 combined with CEA and CA153 was 0.897 and 0.903, respectively, while the AUC of KL-6 combined with both of them was 0.902. The diagnostic value of the combination of all three was superior to CEA alone (*p* = 0.001) but was equivalent to KL-6 (Figure 2).

### 3.2. Association of KL-6 with CEA and CA153

**Figure 3:**
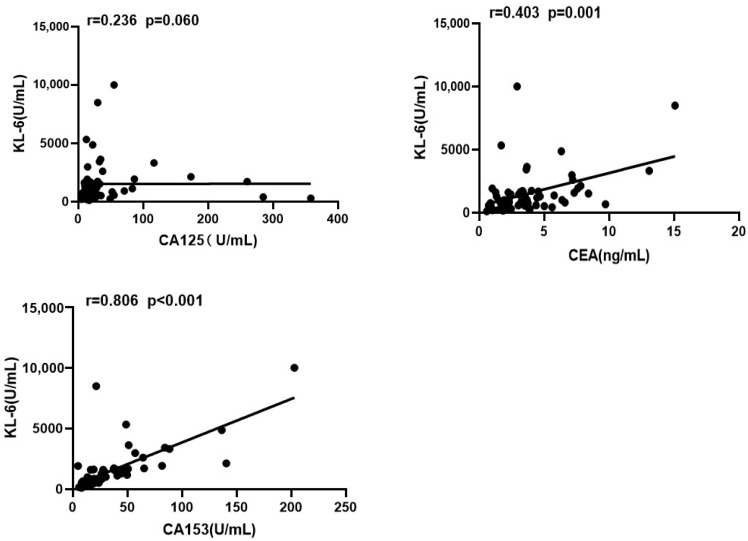
CEA and CA153 were correlated with KL-6; CA125 was not correlated with KL-6. r-correlation index; p-probability.

Results of Spearman’s correlation analysis revealed that KL-6 levels were positively correlated with CEA (r = 0.403, *p* = 0.001) and CA153 (r = 0.806, *p* < 0.0001); however, there was no significant correlation between KL-6 and CA125 (r = 0.236, *p* = 0.060) (Figure 3).

### 3.3. Association of KL-6 and Tumor Markers with Disease Severity

Lung function represents the severity of ILD; in particular, a decrease in FVC%, TLC%, and DLCO% often indicates a poor prognosis. This study found that KL-6, CEA, and CA153 were negatively correlated with FVC%, FEV1%, TLC%, and DLCO% (all *p* < 0.05) (Table 2). In addition, KL-6 was not associated with smoking or age.

### 3.4. Analysis of Potential Risks for pSS-ILD

The results of single-factor logistic regression analysis are showed in Table 3 and Table 4. Single-factor analysis showed that KL-6, CEA, CA153, CA125, FVC%, TLC%, and DLCO% were potential risk factors for pSS-ILD, whereas age, sex, and smoking were not associated with pSS-ILD. Multivariate analysis showed that KL-6 and CA125 were more statistically significant. However, KL-6 was significant compared with the non-ILD group and control group (Table 5 and Table 6).

## 4. Discussion

Via a comprehensive study, we found that the KL-6 and serum tumor biomarkers were elevated in patients with pSS-ILD compared with non-ILD and healthy controls in this study. Multi-variable and ROC analysis revealed a significant association between KL-6, CA125, and pSS-ILD.

The pathogenesis of pSS-ILD is not fully understood, and the time of onset of ILD with respect to pSS diagnosis is variable; for example, while 10–51% of ILD can present before the diagnosis of pSS [20], ILD can also occur at or after pSS diagnosis [21]. The variable onset of ILD in patients with pSS makes it challenging to understand the disease process. HRCT is an important diagnostic method for ILD. However, some patients present without or with mild respiratory symptoms, which is especially common in elderly patients who may only suffer from dry mouth and dry eye symptoms. HRCT is expensive and somewhat radioactive, so it is difficult to implement early in patients who lack typical respiratory symptoms. Therefore, in the absence of clinical suspicion of ILD, the diagnosis may be delayed. Early diagnosis and treatment are important to improve the quality of life of patients and reduce the burden on healthcare systems and society. It is particularly important to find a non-invasive and convenient method to follow up and monitor such patients. Biomarkers have been shown to be useful in the diagnosis and prognosis of many diseases, and, over the years, many biomarkers have been proposed to play important roles in diagnosis, prognosis, and treatment decisions [22]. Blood biomarkers are minimally invasive and easy to detect. More and more articles have reported that combinations of biomarkers may be useful tools for screening ILD and predicting prognosis [23,24]. We compared KL-6 levels in 1084 subjects consisting of ILD, non-ILD pulmonary diseases, and healthy individuals previously. Serum KL-6 levels was found to be a promising diagnostic biomarker for ILD in Chinese patients [7]. This diagnostic value was also found in our previous study conducted in IPF patients [9]. Bao Y et al. [25] found a positive correlation between serum tumor marker levels and CTD-ILD. Higher levels of CA153 and CYFRA21-1 indicate an increased risk of ILD and may be useful biomarkers for detecting the occurrence of CTD-ILD. KL-6 is a glycoprotein with a high molecular weight that is produced mainly by damaged or regenerated alveolar type II lung cells. Later studies demonstrated that elevated KL-6 levels were associated with ILD, especially IPF and CTD-ILD. KL-6 can reflect ILD severity, monitor disease progress, and has certain advantages in disease diagnosis [12,26,27]. Based on the above understanding of tumor markers and KL-6, we focused on the relevant literature on KL-6 and tumor markers and carried out a study on the clinical diagnostic value of KL-6 and tumor markers in pSS-ILD.

The results of serum biomarker detection showed that the serum levels of KL-6, CEA, CA125, and CA153 in pSS-ILD patients were significantly higher than those in the non-ILD group and healthy control group (*p* < 0.05). The result suggests that KL-6 and tumor markers might be related to the development of ILD in pSS. This result was consistent with previous studies. Weng et al. [28] compared the KL-6 levels in 69 pSS patients—among them, 59 complicated with ILD and 10 without—and found that KL-6 was one of the factors associated with the presence of ILD in patents with pSS. Serum KL-6 levels might also be a useful biomarker in predicting the prognosis of patients with pSS-ILD [29]. Zheng et al. [30] conducted a retrospective study in patients with RA and found that serum KL-6 and tumor marker (CEA, CA153, and CA19-9) levels were elevated in patients with RA-ILD and were associated with ILD severity, supporting the clinical value of these markers as disease-related biomarkers. Moreover, Shi et al. [18] conducted a study in 168 patients with pSS-ILD and 538 age- and sex-matched pSS-non-ILD patients and found that serum levels of tumor markers were elevated in pSS-ILD patients compared with pSS-non-ILD patients. However, CA153 was the only tumor marker related to ILD with AUC of > 0.7 (AUC = 0.743, 95% CI: 0.70–0.79). Our study also discovered that CA199 was not increased in pSS-ILD. The mechanism was still unclear and needed to be further confirmed by our prospective study.

Our further findings suggest that the AUC of KL-6, CEA, and CA153 were 0.902, 0.723, and 0.837. However, the AUC of KL-6 combined with CEA and CA153 was 0.897 and 0.903, respectively. The results found that KL-6 had a good correlation with pSS-ILD. It also further indicated that tumor markers combined with KL-6 were more valuable in the diagnosis of pSS-ILD.

Our study also found a correlation between KL-6 and CA153 and CEA, and the correlation between the two had also been proposed in the related literature that had been published [31]. In the future, whether they can be replaced by each other needs further research to confirm.

Lung function is an indicator of ILD severity. Hu C et al. [32] found that serum KL-6 levels were negatively correlated with lung function tests (*p* < 0.01), including FVC%, TLC, and DLCO%. Balestro et al. [33] found that the level of CA199 was negatively correlated with ΔFVC/year (r = −0.261; *p* = 0.03); this association was observed in patients with rapidly progressing IPF (r = −0.51; *p* = 0.005). Furthermore, CA199 may be a marker of disease severity in patients with end-stage ILD, especially those with rapid IPF progression, and its level was inversely associated with functional decline. Our findings revealed that CEA and CA53 were correlated with KL-6, and KL-6, CEA, and CA153 were negatively correlated with FVC%, TLC%, and DLCO%, thereby indicating the correlation of these parameters with disease severity.

In our study, we found that serum KL-6 level and tumor markers (CEA, CA125, CA153) were significantly higher in patients with pSS-ILD than in those with non-ILD as well as healthy controls, but multivariate analysis KL-6 was more abnormally increased and could be used to identify ILD development in patients with pSS. These are consistent with the findings of previous CTD-ILD related studies [34,35,36].

We also found the sequence of occurrence of pSS and ILD. A total of 68.7% of ILD occurred before pSS diagnosis, 21.9% of ILD occurred simultaneously with pSS, and 9.4% of ILD occurred after pSS diagnosis. However, the mechanism is not clear, which is something we need to further study in the future.

There were several potential limitations. Firstly, this is a single-center design, which might result in selection bias and influence the results. Secondly, this is a cross-sectional study; the sequential order of pSS and ILD could not be known. Further prospective multicenter studies are warranted to address these limitations and improve our understanding of the clinical utility of KL-6 in patients with pSS-ILD.

In conclusion, we evaluated the association between clinical values of KL-6, tumor markers, and ILD in patients with pSS and found that KL-6 and tumor markers such as CEA, CA153, and CA125 in patients with pSS-ILD were higher than that in patients with non-ILD, but KL-6 was more statistically significant and could be used to identify ILD development in patients with pSS. KL-6 might be a useful and noninvasive biomarker for pSS-ILD and has great potential for future clinical application.

## Figures and Tables

**Table 1:** Demographic and clinical characteristics of patients in the pSS-ILD, non-ILD, and healthy control groups.

Characteristics	pSS-ILD	non-ILD	H
	n = 64	n = 23	n = 45
Sex (M/F)	22/42	9/14	10/35
Age (y)	62.0 (53.0, 66.8)	62.0 (49.0, 68.0)	56 (54.0, 60.5)
Smoking			
Smoker n (%)	20 (31.2)	10 (43.5)	9 (20.0)
Non-smoker n (%)	44 (68.8)	13 (56.5)	36 (80.0)
CEA (ng/mL)	3.1 (1.8, 4.6) *	1.8 (1.3, 2.8)	1.7 (1.1, 2.7)
CA19-9 (U/mL)	13.5 (7.3, 30.8)	10.6 (5.8, 16.5)	9.9 (6.4, 19.8)
CA125 (U/mL)	18.0 (12.1, 32.9) *	10.3 (8.9, 17.5)	9.0 (7.2, 13.2)
CA153 (U/mL)	23.9 (15.2, 42.3) *	8.7 (6.3, 13.8)	11.7 (7.9, 16.8)
IgG (mg/dL)	1445 (1155, 1725) #	1190 (1040, 1410)	
KL-6 (U/mL)	995.5 (565.5, 1647.3) *	232 (169.0, 368.0)	240 (171.0, 342.5)
FEV1%	84.4 (65.0, 95.1)	88.0 (78.0, 101.5)	
FVC%	82.5 (64.4, 97.3)	99.0 (87.0, 115.0)	
TLC%	71.2 (63.9, 87.3)	90.1 (72.0, 99.8)	
DLCO%	63.0 (45.9, 69.8) *	76.0 (71.0, 87.5)	

Note: Variables with skewed distribution are presented as median (interquartile range IQR). *: *p* < 0.01 (compared with the non-ILD or HC group); #: *p* < 0.05 (compared with the non-ILD group). CEA-carcinoembryonic antigen; CA19-9-carbohydrate antigen 19-9; CA125-carbohydrate antigen 125; CA153-carbohydrate antigen 153; IgG-Immunoglobulin G; KL-6-Krebs von den Lungen-6; FEV1%-forced expiratory volume in 1 s; FVC%-forced vital capacity; TLC%-total lung capacity, and DLCO%-diffusing capacity for carbon monoxide.

**Table 2:** The correlation between pulmonary function tests, KL-6, and tumor markers in patients with pSS-ILD.

	FVC%		FEV1%		TLC%		DLCO%	
	r	*p*	r	*p*	r	*p*	r	*p*
KL-6	−0.395	0.001	−0.264	0.035	−0.506	<0.001	−0.484	<0.001
CEA	−0.384	0.002	−0.276	0.027	−0.404	0.001	−0.323	0.009
CA153	−0.396	0.001	−0.274	0.028	−0.479	<0.001	−0.388	0.002

**Table 3:** Single-factor logistic regression analysis of factors associated with pSS-ILD compared with non-ILD patients.

Variables	OR	95% CI	*p* Value
KL-6	1.005	(1.002, 1.007)	<0.001
CEA	1.758	(1.174, 2.634)	0.006
CA125	1.026	(0.992, 1.061)	0.134
CA199	1.041	(0.992, 1.092)	0.105
CA153	1.121	(1.045, 1.201)	0.001
FVC%	0.968	(0.945, 0.992)	0.009
TLC%	0.964	(0.937, 0.993)	0.014
DLCO%	0.935	(0.900, 0.971)	<0.001
Age	0.995	(0.949, 1.043)	0.832
Smoking	1.692	(0.636, 4.506)	0.292
Sex	0.815	(0.305, 2.179)	0.683

CI: confidence interval, OR: odds ratio.

**Table 4:** Single-factor logistic regression analysis of factors associated with pSS-ILD compared with the healthy population.

Variables	OR	95% CI	*p* Value
KL-6	1.009	(1.005, 1.014)	<0.001
CEA	1.619	(1.213, 2.159)	0.001
CA125	1.180	(1.086, 1.283)	<0.001
CA199	1.026	(0.997, 1.056)	0.084
CA153	1.135	(1.066, 1.207)	<0.001
Age	1.003	(0.961, 1.047)	0.873
Smoking	0.550	(0.223, 1.355)	0.194
Sex	1.833	(0.767,4.384)	0.173

**Table 5:** Multinational logistic regression analysis of factors associated with pSS-ILD compared with non-ILD patients.

Variables	OR	95% CI	*p* Value
KL-6	1.005	(1.002, 1.007)	<0.001

**Table 6:** Multi-variable logistic regression analysis of factors for pSS-ILD compared with the healthy population.

Variables	OR	95% CI	*p* Value
KL-6	1.010	(1.005, 1.014)	<0.001
CA125	1.126	(1.017, 1.248)	0.023

Note: Logistic input method was used for statistical methods.

## Data Availability

The datasets generated and/or analyzed during the current study are not publicly available but are available from the corresponding author on reasonable request.

## Abbreviations

pSS	Primary Sjögren’s syndrome
ILD	Interstitial lung disease
KL-6	Krebs von den Lungen-6
CEA	Carcinoembryonic antigen
CA153	Carbohydrate antigen 153
FVC	Forced vital capacity
FEV1	Forced expiratory volume in 1 s
TLC	Total lung capacity
DLCO	Diffusing capacity for carbon monoxide
CTD-ILD	Connective tissue disease-related interstitial lung diseases
HRCT	High-resolution CT
IPF	Idiopathic pulmonary fibrosis
IQR	Interquartile range
ROC	Receiver operating characteristic
IgG	Immunoglobulin G
